# Empirical antifungal therapy with an echinocandin in critically-ill patients: prospective evaluation of a pragmatic Candida score-based strategy in one medical ICU

**DOI:** 10.1186/1471-2334-14-385

**Published:** 2014-07-11

**Authors:** Rémi Bruyère, Jean-Pierre Quenot, Sébastien Prin, Frédéric Dalle, Clara Vigneron, Serge Aho, Cristobal Leon, Pierre-Emmanuel Charles

**Affiliations:** 1 Service de Réanimation Médicale, Hôpital Bocage Central, CHU Dijon, 14 rue Gaffarel, BP 77908-21079, Dijon, Cedex, France; 2 Laboratoire de Mycologie, Plateau Technique de Biologie, CHU Dijon, Dijon, France; 3 Service d’Epidémiologie et d’Hygiène Hospitalière, Hôpital Bocage Central, CHU Dijon, 14 rue Gaffarel, BP 77908-21079, Dijon, Cedex, France; 4 Intensive Care Unit, Hospital Universitario de Valme, Universidad de Sevilla, Sevilla, Spain

**Keywords:** Invasive candidiasis, Candidemia, Candida sp, Sepsis, Echinocandin, Nosocomial infection

## Abstract

**Background:**

Invasive candidiasis (IC) is a life-threatening ICU-acquired infection. A strong correlation between time to antifungal therapy (AFT) administration and outcome has been established. Empirical therapy benefit should be balanced with the risk of echinocandin overuse. We assessed therefore a decision rule that aimed at guiding empirical therapy.

**Methods:**

A 45-month prospective cohort study in a teaching medical ICU. All of the patients with suspected IC (uncontrolled sepsis despite broad spectrum antibiotics without any bacterial proven infection in patients with Candida score ≥ 3 points including multifocal *Candida* sp. colonization) were eligible. The primary endpoint was proven IC diagnosis (i.e., candidemia) following treatment onset. Timing of AFT administration was also investigated in those latter patients. Antifungal therapy step-down and discontinuation was done according to international guidelines in patients with candidemia. Otherwise, echinocandin discontinuation was encouraged in patients without proven IC, excepting when a clinical improvement was achieved without any other explanation that antifungals initiation (i.e., probable IC). In addition, a survival multivariate analysis using a Cox model was conducted.

**Results:**

Fifty-one patients were given an echinocandin with respect to our decision rule. Among them, candidemia was diagnosed thereafter in 9 patients. Over the same period, antifungal therapy was triggered by candidemia announcement (i.e., definite therapy) in 12 patients who did not fulfill criteria for empirical therapy before. Time elapsed from candidemia onset to echinocandin therapy initiation was shortened (0.4 [0.5] vs. 2.4 [2.8] hours; p = 0.04) when it was given empirically. In addition, 18 patients clinically improved under empirical antifungal therapy without any obvious other explanation, despite IC remained unproven. Moreover, echinocandin exposure duration was independently related to survival in those patients. Over the same period, our predefined criteria for empirical therapy were overruled in 55 cases. None of them develop IC thereafter. Finally, Our decision rule allowed IC early recognition of proven/probable IC with sensitivity, specificity, positive and negative predictive value of 69.2%, 82.1%, 69.2% and 82.1%, respectively.

**Conclusion:**

Implementation of pragmatic guidelines for empirical AFT based on CS and fungal colonization assessment could be useful in selecting patients who really benefit from an echinocandin.

## Background

Given the high mortality rate attributable to invasive candidiasis (IC) and the lack of reliable diagnosis tools, antifungals are often started in high-risk patients with severe sepsis or septic shock despite the absence of proven disease. According to current guidelines, echinocandins are the drugs of choice in this setting [1,2]. However, the level of evidence supporting empirical therapy is low given the lack of conclusive randomized controlled trials so far [3]. In addition, the early identification of patients who will really benefit from antifungal therapy (AFT) remains a matter of concern, since any delay in administering an appropriate antifungal drug is likely to worsen the outcome of such patients [4-8]. It is also known that blood cultures can provide false negative results given their lack of sensitivity [9].

In an attempt to improve our skills in diagnosing IC, clinical rules were derived from observational studies and some of these were then tested in large cohorts of ICU patients [10-13]. Among these rules, the Candida Score (CS) is one of the most popular. Although calculating the CS requires the results of fungal cultures of at least two distinct body sites, it is easy to use at the bedside. The CS, however, has a low positive predictive value (23%), while its negative predictive value can reach 99% if the 3-point threshold is used. Such a “risk-factor-driven” strategy would be of potential interest, at least to identify patients in whom IC is very unlikely. As a result, applying the CS in the everyday practice could lead to the overuse of antifungals, thereby leading to problems of cost and toxicity and ecological issues. Thus, it has recently been reported that previous exposure to echinocandins was independently associated with the risk of decreased susceptibility of *Candida* sp. [14]. Moreover, no studies that aimed to assess the CS as a trigger for antifungal therapy in the ICU have been reported so far, while a “sepsis-driven strategy” was recently evaluated in a randomized controlled trial [3]. This study included critically-ill patients who all presented with persistent fever despite broad-spectrum antibiotics. No difference in outcome was reported whether they received fluconazole or placebo. Obviously, *Candida* sp. colonization was infrequent in the included patients. As a result, this “sepsis-driven” strategy might lead to unnecessary and even harmful antifungal treatments in this low-risk cohort of patients.

Biomarkers of fungal infection, including (1,3)-ß-D-glucan assay and *Candida* sp. DNA detection, are of growing interest since they could allow earlier identification of IC [15]. However, there have been few prospective interventional studies and these lab tests are costly and not yet routinely available in many hospitals. To select the patients in whom performing such tests will be really useful remains challenging so far. The time is therefore ripe to develop clinically-relevant decision rules and antifungal-stewardship programs [16].

We thus conducted a prospective study to assess the clinical relevance of a pragmatic decision rule based on the combination of a “risk-factor-driven” (i.e., Candida Score), and the “sepsis-driven” strategy (uncontrolled sepsis despite broad-spectrum antibiotics) in our 15-bed ICU. Our main goal was to evaluate the ability of this strategy to identify patients likely to benefit from early antifungal therapy reliably and promptly before IC became obvious.

## Methods

### Study population and design

We conducted a prospective study between 1^st^ January 2008 and 15^th^ August 2011 in our 15-bed medical ICU.

We included all patients in whom antifungal treatment with an echinocandin was started after new local guidelines were implemented in our ICU.

Our guidelines were based on the recommendations from the IDSA, available since September 2007 (47^th^ Interscience Conference on Antimicrobial Agents and Chemotherapy, 2007), and enriched by published data regarding the assessment of the most relevant risk factors for IC in the ICU, including the Candida score [1,2,11]. Briefly, antifungals were started to patients with either proven (“definite therapy”) or suspected infection (“empirical therapy”) as described above. Considering that critically-ill patients should all be considered “unstable”, echinocandins (either caspofungine or micafungine) were the preferred drugs. Antifungal prophylaxis was never recommended. In accordance with French law and the Helsinki Declaration, no informed consent was required since all measurements were part of routine management, as confirmed by our local Ethics Committee (Comité de Protection des Personnes Nord-est).

#### “Definite therapy”

Every patient with at least one positive blood culture was given an echinocandin. The treatment was started as soon as the attending physician was informed by telephone that the blood culture was positive. Treatment duration and the decision for step-down therapy with fluconazole, provided the isolated yeast was covered by azoles, were dependent on the judgment of the physician in charge in accordance with international guidelines. Every vascular catheter was removed within a 24-hour time window following the first administration of antifungals.

#### “Empirical therapy”

Every patient hospitalized in the ICU with uncontrolled sepsis (defined as either persistent fever, volume expansion requirement > 1000 ml/day or inability to decrease the need for vasoactive support) despite at least one 2-day course of antibiotics active against any bacterial pathogen (except coagulase-negative *Staphylococci*) isolated from a relevant site or with no proven concurrent bacterial infection, were considered for antifungal therapy if the CS was equal to or more than 3 points. Importantly, as multifocal colonization with *Candida* species is part of the CS, the rules require the implementation or at least the continuation (i.e., if not available the day IC was suspected) of empirical therapy. For this purpose, since surveillance fungal cultures were not routinely performed in our ICU, clinical samples other than blood culture (BC) (i.e., skin, tracheal aspirates, rectal swab, urine and gastric juice) if not yet available were obtained the day IC was suspected. Antifungals were withheld or withdrawn if no more than one body site was positive for *Candida* sp.

If *Candida* sp. was subsequently identified in BC, the “definite therapy” guidelines were applied.

In the absence of a positive BC for *Candida* sp. within the following 3 days, antifungals were stopped if another pathogen not susceptible to the ongoing antibacterial therapy was finally isolated from any relevant site and the patient was excluded from the analysis. Otherwise, echinocandin continuation was encouraged only when a clinical improvement was achieved without any other explanation that antifungals initiation.

Importantly, the attending physician (PEC and RB) checked daily whether all of the above mentioned criteria regarding empirical therapy were fulfilled. Otherwise, treatment continuation was strongly discouraged.

In accordance with French law, no informed consent was required since all measurements were part of routine management, as confirmed by our local Ethics Committee.

### Definitions

Invasive candidiasis was considered proven according to standard definitions.

One episode of ICU-acquired candidemia was defined as the recovery of *Candida sp*. in one or more blood cultures drawn more than 2 days after admission. The onset of candidemia was defined as the day the first positive blood culture was obtained.

Probable IC was defined as the development of severe sepsis/septic shock according to current definitions in one high-risk patient as described above with a significant clinical improvement under antifungal therapy (i.e., SOFA score decrease within the first 5 days of therapy) without any other relevant explanation.

### Measurements

Baseline characteristics (age, gender, type of admission, main comorbidities, severity of the illness on admission assessed by the Simplified Acute Physiology Score II [SAPS II]) of each included patient were collected. Clinical and biological follow-up was performed daily for one week once antifungal therapy had been started.

Besides the usual physiological parameters, the procalcitonin (PCT, Kryptor® immunoassay, Thermofisher, Hennigsdorf, Germany), the white blood cells count (WBC), the blood lactate and the SOFA score were monitored daily [17].

In every patient with suspected infection, blood cultures using both classical medium and Mycosis® (Becton Dickinson®) were prepared, and samples from relevant sites were also taken and cultured. Multifocal colonization with *Candida* sp. was also assessed as previously described.

Bacterial infections were diagnosed by the attending physician according to current definitions and prospectively recorded.

### Endpoints and outcomes

The included patients were retrospectively classified as having proven-probable IC or no IC according to the above-mentioned definitions. Those in whom IC was diagnosed before any empirical therapy was started over the same period were considered false negatives according to our clinical rule provided they did not fulfill the inclusion criteria the day the first positive blood sample was drawn.

Timing to AFT administration was measured in the only patients with candidemia.

Both ICU and in-hospital mortality among patients groups were considered for outcome assessment.

### Statistical analysis

Values are expressed as mean ± standard deviation (SD) unless otherwise stated. Continuous variables were compared using the Mann Whitney *U* test. Categorical variables were compared using the chi-square test.

Sensitivity, specificity, positive and negative predictive values of our prescription rule for early recognition of proven and/or probable IC was calculated according to standard definitions. In the first set of analyses, the gold standard for the diagnosis of IC was *Candida* sp. positive BC as defined above. Secondly, we assessed the accuracy of our clinical diagnosis rules by considering proven and probable IC as a whole.

Then, survival analysis was performed in an attempt to determine to whish extent echinocandin exposure duration could be protective in the patients with suspected IC regardless of subsequent microbiological confirmation (e.g., candidemia), as previously shown in patients included into randomized controlled trials [18]. A multivariate analysis based on a Cox model was used to eliminate potential confounders. In addition to the duration of treatment with echinocandin once IC was suspected or proven, covariates found to be associated with ICU mortality by univariate analysis (haematological malignancy, SOFA score the day antifungals were started, renal replacement therapy [RRT]) were considered for being entered into the model. However, given the low number of events (i.e., death in the ICU: n = 26), no more than 2 variables could be thus tested in addition to echinocandin therapy duration. The choice of such variable was based on the results of a Classification and Regression Tree (CART) analysis [19].

A *p* value ≤ 0.05 was considered statistically significant for all analyses. Statview software was used for most of the analyses (SAS Institute, Cary, NC, USA). Remaining analysis including ROC curves construction and CART analyses were performed with the STATA statistical package (College Station, Tx, USA).

## Results

### Study population

**Figure 1:**
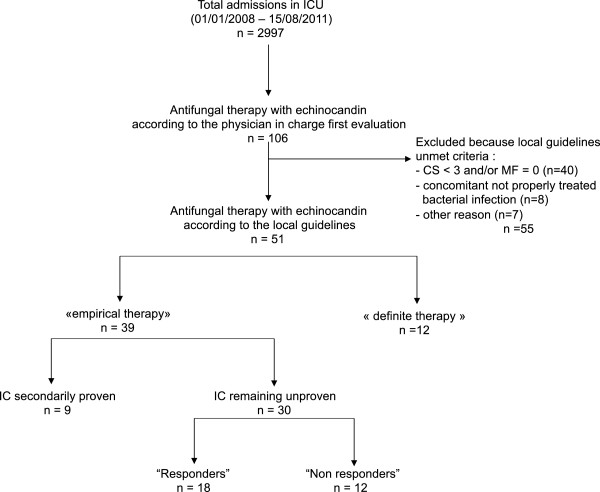
**Flowchart of selection of study patients.** ICU: intensive care unit; ATF: antifungals; IC: invasive candidiasis; CS: candida score; MF: multifocal colonization.

During the study period, 2997 patients were admitted to our ICU (Figure 1). One hundred and six (3.5%) received at least one dose of echinocandin. Among these, 94 were considered for empirical antifungal therapy according to the attending physician’s first evaluation. After completion of the above-described IC risk-factor assessment, 39 (41.5%) patients met the inclusion criteria and therefore remained on antifungal therapy. Conversely, antifungal drugs were withdrawn in 55 patients (CS < 3: n = 40; concomitant not properly treated bacterial infection: n = 8; other reason: n = 7).

Over the same period, definite therapy with an echinocandin was started in 12 patients once the physician in charge had been told that the blood culture for *Candida* sp. was positive.

The main baseline characteristics of both subsets of patients are presented in Table 1. Obviously, the two groups were not significantly different.

**Table 1:** Baseline characteristics of the patients receiving antifungal therapy with an echinocandin according to local guidelines

		**Empirical therapy (n = 39)**	**Definite therapy (n = 12)**	***p***
Age (years)		65.9 (13.3)	64.6 (17.5)	0.77
Gender (men,%)		25 (64.1)	7 (58.3)	0.72
SAPS II		49.1 (17.1)	49.8 (8.1)	0.89
Admission diagnosis				0.17
	Sepsis (N. [%])	20 (51.2)	4 (33.3)	
	Respiratory distress (N. [%])	15 (38.4)	8 (66.6)	
	Other (N. [%])	4 (10.2)	0 (0)	
Underlying disease(s)				
	Diabetes mellitus (N. [%])	12 (30.7)	1 (8.3)	0.11
	Cardiac failure (N. [%])	15 (38.4)	2 (16.6)	0.16
	Chronic lung disease (N. [%])	12 (30.7)	1 (8.3)	0.11
	Cirrhosis (N. [%])	5 (12.8)	3 (25)	0.31
	Chronic renal failure (N. [%])	3 (7.7)	1 (8.3)	0.94
	Cancer (N. [%])	6 (15.4)	1 (8.3)	0.53
	Hemopathy (N. [%])	3 (7.7)	0 (0)	0.32
	Immunosuppression (N. [%])	9 (23.1)	1 (8.3)	0.26
	Transplantation (N. [%])	3 (7.7)	1 (8.3)	0.94

SAPS II: simplified acute physiologic score II.

### Clinical relevance and diagnosis accuracy of local guidelines

None of the 55 patients in whom empirical therapy was stopped because they did not meet our decision rule criteria were diagnosed with IC thereafter. As a result, they were considered true negatives.

**Figure 2:**
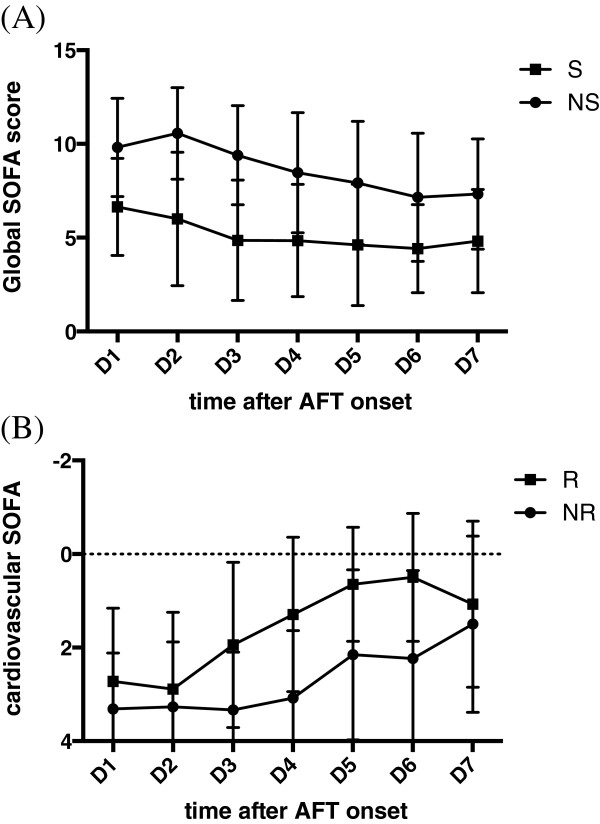
**Organ failure kinetic expressed as global SOFA score value (panel A) and the cardiovascular SOFA score (panel B) daily measurements over the first week of treatment in the subset of patients (n = 30) who received empirical echinocandin therapy according to local guidelines without any proven IC thereafter.** The patients were classified as “responders” if SOFA decreased by more than 1 point during the first 5 days of therapy (n = 18). Otherwise, they were considered as “non responders” (n = 12). D: day; SOFA: sequential organ failure assessment; IC: invasive candidiasis; R: responders; NR: non responders.

In contrast, among the 39 patients who were given empirical antifungal therapy with an echinocandin in accordance with our decision rule, IC was subsequently proven, as candidemia was diagnosed, in 9 (23.1%) cases. As expected, the time between drawing the first positive blood sample and the implementation of AFT was significantly shorter in patients who received empirical therapy than in those whose treatment was triggered by a positive blood culture (i.e., “definite therapy” group) (0.4 [0.5] vs. 2.8 [0.8] days; p < 0.01) (Table 2).Moreover, a clinical response as defined above was reported in 18 (60.0%) of the 30 remaining patients even though IC was not proven (i.e., probable IC). Of note, apart from organ failure, the resolution of hemodynamic instability, as assessed by the SOFA score scale, was the most striking feature in this group (Figure 2). In contrast, 12 (40.0%) patients did not improve under antifungal therapy. They were therefore considered false positives.

**Table 2:** Description of suspected or proven invasive candidiasis episodes and outcomes in patients receiving antifungals as either empirical or definite therapy according to local guidelines

	**Empirical therapy (n = 39)**	**Definite therapy (n = 12)**	***p***
Time elapsed between ICU admission and AFT (days)	8.8 (9.9)	9.0 (6.8)	0.96
Time between BC and AFT (days)*	0.4 (0.5)	2.8 (0.8)	<0.01
AFT duration (days)	9.2 (6.0)	9.1 (6.4)	0.95
Echinocandin therapy duration (days)	6.7 (4.7)	5.7 (4.2)	0.54
Fluconazole therapy duration (days)	2.6 (5.1)	3.4 (5.2)	0.62
Septic shock (N. [%])		31 (79.5)	6 (50.0)	0.04
RRT (N. [%])		22 (56.4)	4 (33.3)	0.16
SOFA D1		8.8 (3.0)	8.4 (3.0)	0.14
PCT D1 (pg/L)		3.6 (6.6)	4.2 (10.6)	0.56
Proven bacterial infection prior to AFT (N. [%])		14 (46.7)	9 (42.8)	0.79
Outcomes Length of ICU stay (days)		21.4 (15.7)	25.6 (22.4)	0.47
MV duration (days)		15.7 (13.3)	22.4 (24.7)	0.22
Vasoactive support duration while receiving AFT (days)		3.8 (2.9)	2.3 (2.6)	0.11
ICU mortality (N. [%])		20 (51.2)	6 (50.0)	0.93
In-hospital mortality (N. [%])		25 (64.1)	9 (75.0)	0.14

*including the only patients with candidemia.

ICU: intensive care unit; AFT: antifungal therapy; BC: blood culture; MV: mechanical ventilation; SOFA: sequential organ failure assessment; PCT: procalcitonin; RRT: renal replacement therapy.

Over the same period, 12 candidemia episodes were detected before any antifungal therapy was started. It is worth noting that none of these patients met all of the required criteria on the day the first positive blood sample was drawn (Candida score < 3 points: n = 4; lack of multifocal colonization: n = 6; concomitant not properly treated bacterial infection: n = 4). These patients were therefore considered false negatives according to our guidelines about empirical therapy.

As a result, the accuracy of our clinical rules regarding the early identification of proven IC was 42.9%, 64.7%, 23.1%, 82.1% for sensitivity, specificity, PPV and NPV, respectively (Table 3). The corresponding AUROCC was 0.54. When probable and proven IC were considered as a whole, sensitivity, specificity, PPV and NPV were 69.2%, 82.1%, 69.2% and 82.1%, respectively. Although sensitivity was lower, our guidelines were more accurate than the Candida score alone in predicting the risk of proven or probable IC in terms of specificity and positive predictive value. However, the AUROCCs were similar (0.75 vs. 0.76, respectively).

**Table 3:** Diagnosis accuracy of the Candida Score regarding the risk of proven and/or probable IC, as compared with our local guidelines

	**Sensitivity (95% CI)**	**Specificity (95% CI)**	**Positive predictive value (95% CI)**	**Negative predictive value (95% CI)**	**Positive likelihood ratio**	**Area under the ROC curve (95% CI)**
Candida score & Proven IC	81.0% (58.1–94.5)	47.1% (36.1–58.2)	27.4% (16.8–40.2)	90.9% (78.3–97.5)	1.53	0.64 (0.51–0.76)
Candida score & Proven + probable IC	89.7% (75.8–97.1)	59.7% (47.0–71.5)	56.4% (43.3–69.0)	90.9% (78.3–97.5)	2.23	0.75 (0.65–0.84)
Local guidelines & Proven IC	42.9% (21.8–66.0)	64.7% (53.6–74.8)	23.1% (11.1–39.3)	82.1% (70.8–90.4)	1.21	0.54 (0.40–0.68)
Local guidelines & Proven + probable IC	69.2% (52.4–83.0)	82.1% (70.8–90.4)	69.2% (52.4–83.0)	82.1% (70.8–90.4)	3.86	0.76 (0.66–0.86)

IC: invasive candidiasis; CI: confidence interval; ROC : receiver operating characteristics.

### Microbiological findings

Every case of proven IC was candidemia. A median number of 1 [1-4] BC grew positive. The most frequently isolated species were *C. albicans* (n = 13, 61.9%), *C. glabrata* (n = 5, 23.8%), *C. parapsilosis* (n = 1, 4.8%), *C. inconspicua* (n = 1, 4.8%), *C. tropicalis* (n = 1, 4.8%).

### Outcomes

We then conducted a survival analysis to determine to what extent empirical echinocandin therapy prescribed according to our guidelines could affect patients’ outcomes, since in-hospital mortality was as high as 75% in the definite therapy group.

Our endpoints were both ICU and in-hospital mortality which reached 51.3% and 64.1%, respectively, in this subset of patients. Multivariate analysis based on a Cox model was conducted. In addition to echinocandin therapy duration and the manner antifungals were prescribed (i.e., empiric vs. definite therapy), the selected covariate entered into the model was the use of RRT (CART analysis, see above for details). Thus we showed that the duration of echinocandin therapy and the absence of RRT the day it was started were the only independent predictors of ICU survival in our cohort (Table 4).

**Table 4:** Independent predictors of death in the patients receiving echinocandin as antifungal therapy with respect to local guidelines, according to 3 different Cox analysis models

	**Hazard ratio**	**95% CI**	***p***
Duration of echinocandin therapy	0.88	0.78–0.99	0.05
RRT on D1	3.05	1.29–7.19	0.01
	**Hazard ratio**	**95% CI**	***p***
Empiric therapy (Yes)	1.50	0.57–3.96	0.41
RRT on D1	3.05	1.29–7.19	0.01
	**Hazard ratio**	**95% CI**	***p***
Duration of echinocandin therapy	0.91	0.81–1.01	0.07
Empiric therapy (Yes)	1.19	0.43–3.25	0.74
RRT on D1	3.20	1.29–8.00	0.01

D: day; SOFA: sequential organ failure assessment; RRT: renal replacement therapy; CI: confidence interval.

## Discussion

Invasive candidiasis is a life-threatening infectious complication in the ICU [20]. Although IC is infrequent, empirical antifungal therapy is given to many critically-ill patients presenting with uncontrolled sepsis despite any proof of fungal infection [21]. The clinical evidence in support of this practice is rather weak. However, both American and European experts have recommended empirical antifungal therapy in high-risk patients in the ICU [1,2]. Identifying this subset of patients thus remains a matter of concern [22]. Clinical prediction scores have emerged but their poor positive predictive values could lead to overtreatment with antifungals [15]. As part of an antifungal drugs stewardship program, we therefore decided to build a pragmatic decision rule regarding empirical therapy. Basically, antifungals were given only to patients with a high risk of IC (CS ≥ 3 points) and uncontrolled sepsis despite well-conducted antibiotic therapy for at least 2 days. It was also mandatory to prove multifocal colonization with *Candida* sp. In accordance with international guidelines, echinocandins were the first choice drugs. We show herein that implementing this rule allowed us to provide early therapy to 9 out of 21 patients with proven IC, with a trend towards lower in-hospital mortality (44.4% vs. 75.0%, respectively; *p* = 0.15). On the other hand, treatment was rapidly withdrawn in 55 patients who did not meet our predefined criteria and in whom none developed proven IC thereafter. In addition, we identified a subgroup of 18 patients (46.1% of the whole cohort of high-risk patients as defined by our guidelines without proven IC) in whom we failed to diagnose IC despite the fact that empirical antifungal therapy probably improved the clinical outcome. Finally, we found that in our cohort, the duration of treatment with an echinocandin was independently associated with better survival; regardless drugs were given as empiric or definite therapy.

Identifying patients who will really benefit from empirical antifungal therapy remains a challenge in everyday clinical practice. Many risk factors have been reported and several clinical scoring systems have been proposed and even validated in large cohorts. Among these, the CS is probably the simplest and the most relevant. However, one could criticize its lack of specificity, thus precluding the use of the CS as a decision rule, even though it performs better than other indicators such as the “Pittet index” and “Ostrosky-Zeichner” score, as demonstrated elsewhere [11,23]. To some extent, our findings lend support to the usefulness of the CS. However, the negative predictive value reported herein (90.9%) is lower than previously reported values, which are generally near to 100%. Although the CS has been prospectively validated in a large cohort of critically-ill medical and surgical patients, our findings show that it cannot necessarily be translated into every ICU population in “real life” conditions. Our decision rule proved to be as reliable in our ICU than CS alone, and allowed us to provide early therapy (i.e., before the positive BC) to almost half of the patients who went on to develop IC. This proportion is somewhat greater than those previously reported in observational studies since 6 to 30% rates have been published so far [7,24]. We should, however, admit that further improvements could be expected given the high rates of false positives and false negatives.

Echinocandins are today the most potent drugs against *Candida* sp. (except *C. parapsilosis*) and are thereby recommended as the first-line therapy in critically-ill patients. Actually, compelling evidence from well-conducted randomized controlled trials support the superiority of echinocandins compared with fluconazole or amphotericin B [18]. Thus, it has been shown that the duration of treatment with echinocandins was an independent predictor of survival in the patients with proven IC. Interestingly, we made the same finding in our cohort, and especially in the subset of patients who received empirical therapy. This suggests that treated patients may have benefited from their early antifungal treatment, even though, in some cases, no microbiological evidence of IC was subsequently obtained.

Taken together, our findings suggest that IC could remain hidden in a subgroup of high-risk critically-ill patients, thus illustrating the acknowledged lack of sensitivity of the currently available diagnosis tool, blood culture, in the “medical” setting [25]. Interestingly, this paradigm is even more strongly supported by recently published data, since some authors were able to diagnose IC by the presence of circulating *Candida* DNA in surgical patients [26]. Although speculative, the proportion of such unproven IC is near from 50% in our cohort, matching with these findings.

Our study, however, has several limitations. First and foremost, the small sample size could have led us to miss potential confounding covariates and differences between groups. In particular, as a result, our survival analysis should be interpreted with caution. Moreover, our results were obtained in a single unit and further studies are needed since they may not be relevant elsewhere. In addition, one cannot exclude the possibility that the patients classified as probable IC because of a favorable clinical response to antifungals were in fact suffering from non-documented bacterial sepsis adequately treated by concomitantly administered antibiotics. Actually, the diagnosis of IC in these patients was purely speculative given the lack of microbiological evidence. Unfortunately, we were unable to use non-culture diagnosis methods such as (1,3) ß-D-glucan assay since the test was not yet available in our institution. However, it has been shown that this assay was only as accurate as the clinical response to antifungal in predicting IC in high-risk surgical patients [23,27,28]. Additional studies are therefore needed to determine whether the addition of biomarkers to decision rules like ours could improve the identification of patients likely to develop IC, as proposed elsewhere [15,29,30].

## Conclusion

As a conclusion, our findings suggest that empirical therapy with echinocandin in critically-ill patients with uncontrolled sepsis in medical ICUs could be guided by a clinical decision rule based on risk-factor assessment that includes *Candida* sp. colonization and the CS. Thus, unnecessary treatments could be avoided and almost half of the patients with proven candidemia could receive antifungals promptly, that is to say before a positive BC is obtained. However, since 12 patients out of the 21 with IC did not meet our criteria the day they develop candidemia, the clinical rule presented herein should be considered cautiously. Further improvements are therefore mandatory. Specific serum biomarkers of *Candida* sp. could therefore be helpful.

## Competing interests

The authors declare they have no competing interests.

## Authors’ contributions

PEC and RB designed the study. RB, FD, CV and SP collected the data. PEC drafted the manuscript. PEC and RB performed statistical analysis of the data. CL and JPQ revised the manuscript. All authors read and approved the final manuscript.

## Pre-publication history

The pre-publication history for this paper can be accessed here:

http://www.biomedcentral.com/1471-2334/14/385/prepub
